# Obsessive–compulsive symptoms in psychotic disorders: longitudinal associations of symptom clusters on between- and within-subject levels

**DOI:** 10.1007/s00406-018-0884-4

**Published:** 2018-03-08

**Authors:** Frederike Schirmbeck, Max Konijn, Vera Hoetjes, Mathias Zink, Lieuwe de Haan, For Genetic Risk and Outcome of Psychosis (GROUP)

**Affiliations:** 1 Department of Psychiatry, Academic Medical Centre University of Amsterdam, Amsterdam, The Netherlands; 2 Arkin Institute for Mental Health, Amsterdam, The Netherlands; 3 Central Institute of Mental Health, Medical Faculty Mannheim, University of Heidelberg, Mannheim, Germany

**Keywords:** Psychosis, Obsessive–compulsive, Comorbidity, Longitudinal, Within-subject

## Abstract

Obsessive–compulsive symptoms (OCS) are frequently reported in patients with schizophrenia and have been associated with subjective distress and higher impairment. Recent studies suggest fluctuation in co-occurring OCS and associations with the course of psychotic symptoms. Current evidence is limited by few studies with long assessments intervals and a sole focus on between-subject comparisons. The aim of this study was to specifically investigate co-variation of symptom domains over time within individuals. Patients with a psychotic disorder (*n* = 56) and un-affected siblings (*n* = 49) completed monthly assessments of clinical and subclinical symptoms over 6 months. Mixed-model multilevel analyses examined the variability and relationship between OCS and positive, negative, and depressive symptoms on the between- and within-subject level. Symptom domains were associated across subjects and assessment times, in patients and siblings, with the strongest association between OCS and (subclinical) positive symptoms. Within-subjects, substantial variability and co-variation of all symptom domains was found. Particularly, between-subject differences in positive symptoms and within-subject change in depressive symptoms predicted subsequent OCS in patients 1 months later. This is the first prospective study disaggregating between and within-subject associations between co-occurring OCS and symptom cluster of psychosis. Differences on these two levels suggest different underlying mechanisms. The association between depressive symptoms and subsequent increase/decrease of OCS within patients may have important treatment implications.

**Electronic supplementary material:**

The online version of this article (10.1007/s00406-018-0884-4) contains supplementary material, which is available to authorized users.

## Introduction

Meta-analyses estimate that 12% of patients with schizophrenia also fulfil the criteria for obsessive–compulsive disorder (OCD) and that almost every third (30%) patient reports co-occurring OCS [1, 2]. Recent studies have further shown that patient with primary OCD has an increased risk for a comorbid psychotic disorder [3, 4]. Co-occurring obsessive, intrusive thoughts, and related compulsions are often experienced as severely disturbing and have been found associated with lower subjective wellbeing and quality of life [5–7] and to interfere with successful treatment and social and vocational rehabilitation [7, 8].

Several studies investigated the relationship between OCS and symptom clusters of schizophrenia [9]. A meta-analysis by Cunill et al. concluded that most studies reported more severe global, positive, and negative symptoms [10] if OCS were present. However, some studies found no differences or even less severe psychotic symptoms in patients with OCS co-occurrence [6, 8, 11, 12]. To explain these inconsistencies, authors have referred to methodological reasons such as differences in OCS definition (categorical vs dimensional) or in sample characteristics (e.g., level of severity of OCS or stage of psychotic illness) [7, 10] and the cross-sectional design of these studies. More consistent associations have been reported between OCS in schizophrenia and higher severity of depressive symptoms [7].

To overcome the limitation of cross-sectional designs, longitudinal studies investigated the course and associations between co-occurring symptom domains over time. A study of first episode patients revealed high variability of OCS severity over the 5-year follow-up period. Only a minority reported persistence of OCS, whereas the majority reported either remission of the initial OCS or intermittent OCS. No association was found between OCS occurrence and a more severe course of psychotic symptoms [8]. In contrast, a subgroup of patients with clozapine associated OCS showed stable symptom severity over 12-months [13]. On the categorical level, Cederlöf et al. investigated the association between OCD and schizophrenia and found increased risk for prospective comorbid diagnoses [14]. In a recent longitudinal investigation of patients and siblings of the Genetic Risk and Outcome of Psychosis (GROUP) study, remission of initial OCS was significantly associated with improvement in positive symptoms, emotional distress, and overall functioning. Similar results were found on a subclinical level in siblings [15]. These findings suggest co-variation of severity in different symptom domains. However, data are limited by large intervals of several years between assessments. Furthermore, studies only focused on between-subject associations (symptom interrelation across individuals), but did not investigate within-subject (co)variation. Apart from differences between patients, longitudinal changes and interrelations of symptom clusters within individuals are important to consider to provide treatment options dedicated to improve recovery and quality of life in this patient group [16].

### Aim of the study

The aim of the current study was to investigate the course of psychotic, obsessive–compulsive, and depressive symptoms over time and associations between these symptom clusters on the between- and within-subject level in patients with psychotic disorders and on a subclinical level in siblings. Based on earlier findings [8] we expected to find significant fluctuation in the severity of symptom domains over 6 months. Furthermore, we hypothesized co-variation between the course of OCS and symptoms of psychosis and depression within patients and on a subclinical level within siblings. To explore possible causal inferences, we analysed whether between-subject differences and within-subject changes in psychotic symptoms predicted subsequent change in OCS 4 weeks later and vice versa.

## Materials and methods

### Study design and participants

Patients with a psychotic disorder and un-affected siblings included in the current study, were participants of the GROUP study, who had previously been seen at one of the mental health care institutions belonging to the recruitment site Amsterdam [17]. Participants had agreed to be re-contacted for subsequent research and were asked to take part in the current 6 month follow-up with monthly online assessments of self-rated psychopathology. The procedure of recruitment and population characteristics of GROUP participants have been described in detail elsewhere [17]. In short, inclusion criteria for patients and siblings were (1) age range of 16–50 years and (2) good command of the Dutch language. Patients had to meet DSM-IV-TR criteria for a non-affective psychotic disorder (APA, 2000) as measured with the comprehensive assessment of symptoms and history (CASH) or the Schedules for Clinical Assessment for Neuropsychiatry version 2.1 (SCAN) [18]. An additional inclusion criterion for the sibling group was the absence of a lifetime psychotic disorder.

For the current study, potential participants were contacted by phone, informed about the content and time investment of the study, and asked to participate. All participants provided written informed consent prior to their inclusion in the study, which was approved by the accredited Medical Ethics Review Committee (METC)#NL46405.018.13.

### Assessment instruments

Sociodemographic data on age, gender, education level, age of onset, duration of illness, and medical treatment were collected.

Obsessive–compulsive symptoms (OCS) were assessed with the self-rating obsessive–compulsive inventory-revised (OCI-R; [19]). The OCI-R contains 18 items forming six subscales: Checking, Hoarding, Neutralizing, Obsessing, Ordering, and Washing [20]. Items are answered on a five point Likert scale from not at all (0) to extremely (4). The outcome variable of the current study was the OCI-R total score with a range between 0 and 72.

Positive, negative, and depressive symptoms were measured with the three subscales of the Community Assessment of Psychic Experiences (CAPE; [21]). The CAPE is a 42-item self-report questionnaire, which measures frequency and associated distress with psychotic experiences. The current study used frequency ratings as outcome measures. Items were answered on a four point Likert scale ranging from never (0) to nearly always (3). For both the OCI-R and CAPE, equivalences of the valid and reliable paper–pencil versions to online administration have been documented [22–24].

### Statistical analyses

Sample characteristics at baseline were explored with descriptive statistics.

To investigate whether psychopathology ratings changed over time, fixed-effect regression models were conducted with OCS, positive, negative, and depressive symptoms as the dependent variables and time as the predictor. To account for the hierarchical structure of the data, in which repeated observations were nested within-subjects, and to assess between- and within-subject variability, we included random effects in our analyses.

Raw scores of investigated variables reflect both, how the individuals differ from other individuals and how individuals differ from their usual level. Because we were specifically interested in the association of intra-individual changes, we followed the recommendation of Wang and Maxwell (2015) to disaggregate between- and within-subject components [16]. Therefore, through person-mean centering, we created a between-subject mean component (mean differences between-subjects) and within-subject deviation from this mean (within-subject variability) for all predictor variables. To assess whether variation in positive, negative, and depressive symptoms predicted OCS, we subsequently conducted fixed-effect regression models with OCS as the dependent variable and between- and within-subject variables of positive, negative, and depressive symptoms as predictors. Again, to account for the hierarchical structure of the data, we included random intercepts and random slope in our analyses. To explore possible reciprocal temporal association between symptoms of psychosis and OCS, ‘lead variables’ were created to assess relations to scores 4 weeks later. These were subsequently entered into mixed-model analyses as dependent variables. Data analyses were conducted with the Statistical package for Social Sciences (IBM SPSS Statistics version 24.0, Armonk, NY: IBM Corp.), according to intensive longitudinal methods by Bolger and Laurenceau [25].

## Results

The longitudinal data set consists of 56 patients*6 assessment times = 336 observations (missing data: 3 dropouts at t2, 3 dropouts at t4, 3 individuals missing one assessment = 309 completed assessments) and 49 siblings*6 assessment times = 294 observations (2 dropouts at t2, 1 dropout at t3, 2 individuals missing one assessment = 277 completed assessments). Sample characteristics at baseline and data on overall, between- and within-subject (subclinical) psychopathology, are presented in Table 1.

**Table 1:** Sociodemographic characteristics of patients and siblings at baseline mean and standard deviation of included variables across all six assessments

	Patients mean (SD) *N* = 56	Siblings mean (SD) *N* = 49
Age	35.59 (7.73)	37.41 (9.64)
Gender (male/female)	45/10	19/30
Ethnicity		
Caucasian	48	47
Surinamese	2	0
Mixed	4	2
Education		
Primary school	8	3
Secondary school	12	6
High school	18	11
Vocational education	12	19
University (WO)	6	10
WAIS estimation total IQ	100.43 (15.85)	105.13 (15.86)
Age of Onset First Psychosis	23.23 (7.30)	
Illness duration	12.3 (3.20)	
Medication		
Risperidone	6	
Olanzapine	10	
Quetiapine	4	
Aripiprazole	5	
Clozapine	7	
Haloperidol	2	
Other	2	
Polypharmacy	6	
No medication	14	

Variables	Patients mean (SD) *N* = 309	Siblings mean (SD) *N* = 277
OCI-R		
Overall	9.42 (10.02)	3.25 (3.44)
Between-subject	9.78 (9.42)	3.35 (2.97)
Within-subject	0 (3.36)	0 (1.80)
CAPE positive		
Overall	5.62(8.08)	0.54 (1.11)
Between-subject	5.68 (7.62)	0.54 (0.82)
Within-subject	0 (1.94)	0 (0.72)
CAPE negative		
Overall	9.13 (6.88)	4.17 (3.82)
Between-subject	9.26 (6.28)	4.02 (3.27)
Within-subject	0 (2.90)	0 (1.93)
CAPE depressive		
Overall	5.11 (4.16)	2.93 (2.69)
Between	5.21 (3.70)	2.92 (2.31)
Within	0 (1.76)	0 (1.29)

*OCI-R* obsessive–compulsive inventory-revised, *CAPE* Community Assessment of Psychic Experiences

20 of the 56 patients reported relevant OCS at least once during the assessment period. Based on interpretation guidelines, we defined the presence of relevant OCS as an OCI-R score ≥ 14 [26]. Besides obsessions, participants mainly reported checking, ordering, and hoarding compulsions. Only five siblings reported clinically relevant OCS severity, whereas 19 subjects showed subclinical OCS (OCI > 5).

### Variability in symptom domains over time

Multilevel models of symptom course were conducted with time as the fixed effect and random effects at two levels: at the upper level, the extent to which people vary from the group average (between-subject), and at the lower level, the extent to which individual data-points vary from the individual fitted regression line (within-subject).

Regarding the course of OCS, analysis revealed a significant time effect (*t* = − 2.93, *p* = .005), with decreasing OCS severity. Random effects showed significant between-subject variance for the intercept (*z* = 4.86, *p* < .001) and significant within-subject variability (*z* = 10.05, *p* < .001).

Similar results were found for the three symptom clusters of the CAPE. Analyses showed significant time effects for positive (*t* = − 2.72, *p* = .009), negative (*t* = − 2.76, *p* = .008), and depressive symptoms (*t* = − 3.25, *p* = .002) with decrease in severity over time. Random effects showed significant between-subject variation of intercept in positive (*z* = 5.09, *p* > .001), negative (*z* = 4.69, *p* > .001) and depressive symptoms (*z* = 4.75, *p* > .001). Furthermore, significant between-subject variability in slope was found for positive symptoms (*z* = 2.01, *p* = .045) and depressive symptoms (*z* = 2.05, *p* = .041), as well as significant intercept*slope covariance for these two symptom domains [positive (*z* = − 2.61, *p* = .009), depressive symptoms (*z* = − 2.16, *p* = .031)]. Significant within-subject variability was found in all three symptom clusters (positive: *z* = 10.04, *p* < .001, negative: *z* = 10.06, *p* < .001 and depressive: *z* = 10.07, *p* < .001).

**Fig. 1:**
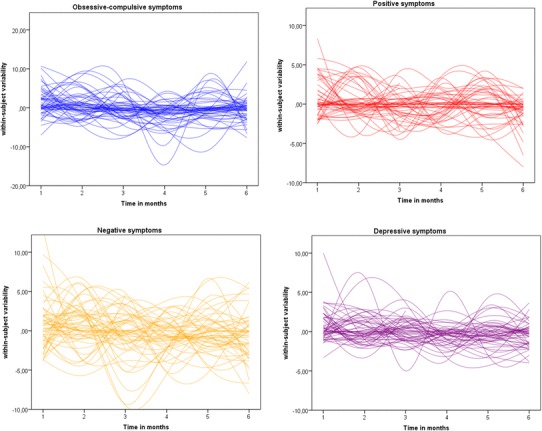
Within-subject change in obsessive–compulsive, positive, negative, and depressive symptoms over 6 months in patients. Legend: obsessive–compulsive (in blue), positive (in red), negative (in orange), and depressive (in purple)

Within siblings, again, significant time effects were found for OCS (*t* = − 3.11, *p* = .003) and CAPE positive symptoms (*t* = − 2.40, *p* = .020). Random effects showed significant between-subject variety in intercepts of all symptom domains (OCS *z* = 4.19, *p* > .001; positive symptoms: *z* = 3.62, *p* > .001; negative symptoms: *z* = 4.07, *p* = < .001; and depressive symptoms: *z* = 3.97, *p* < .001), as well as significant variability in slope for OCS (*z* = 2.74, *p* = .006), positive symptoms (*z* = 2.83, *p* = .005), negative symptoms (*z* = 2.77, *p* = .006) and depressive symptoms (*z* = 2.03, *p* = .042). No significant intercept*slope covariance on the between-subject levels was found. Significant within-subject variability was found in all symptoms domains (OCS: *z* = 9.56, *p* < .001; positive symptoms: *z* = 9.57, *p* < .001; negative symptoms: *z* = 9.55, *p* < .001; and depressive symptoms *z* = 9.55, *p* < .001). Figure 1 displays within-subject variation in symptom domains over time in patients (siblings see supplement Fig. 1).

### Symptom co-variation

**Fig. 2:**
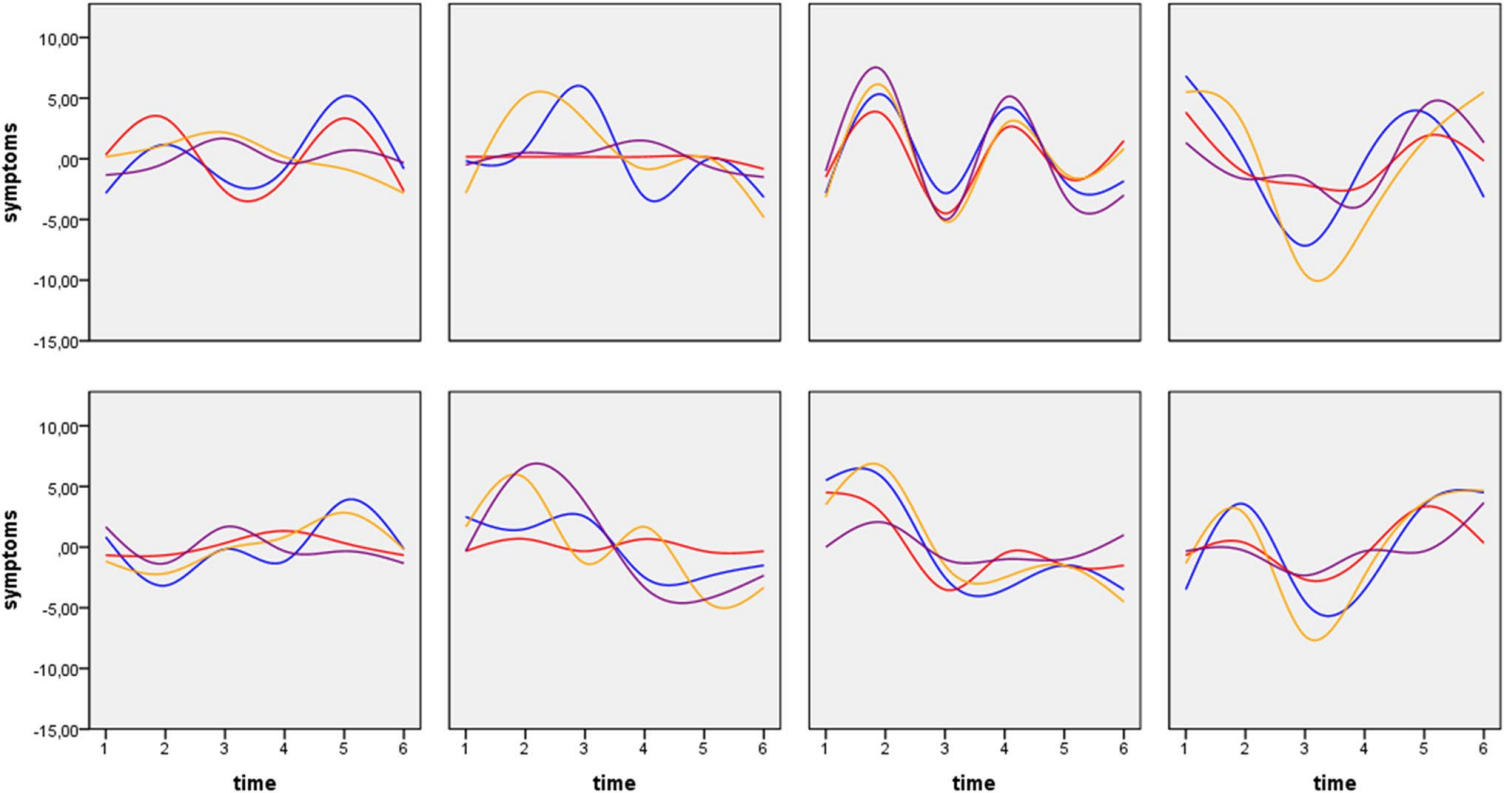
Examples of eight individual panel plots of within-subject (co-)variation in symptom severity in patients. Legend: obsessive–compulsive (in blue), positive (in red), negative (in orange), and depressive (in purple)

To further investigate the association between between-subject differences and within-subject variation in CAPE and co-occurring OCS, the three CAPE variables were disaggregated, as described in “Method”. Figure 2 shows individual panel plots of within-subject symptom co-variation in eight individuals of the patient group (siblings see supplement Fig. 2).

Mixed-model analyses with between-subject and within-subject variables of the CAPE as predictor variables and OCS as measured with the OCI-R as outcome variable were conducted. Because of apparent time trends, we controlled for the effect of time by including the variable as a covariate (‘detrending’) to investigate the relation between predictors and outcome “above and beyond systematic change in the outcome over time” ([27] p. 219) [16]. Results revealed significant associations between OCS and between-subject differences in positive (*t* = 8.89, *p* < .001), negative (*t* = 4.31, *p* < .001) and depressive symptoms (*t* = 5.36, *p* < .001), indicating that subjects who reported more positive, negative, or depressive symptoms also reported more OCS over time. In addition, within-subject variability in all three symptom domains of the CAPE (positive: *t* = 4.71, *p* < .001; negative: *t* = 6.84, *p* < .001; depressive; *t* = 6.27, *p* < .001) were significantly associated with OCS severity, suggesting co-variation.

Including all predictors in one model resulted in a significant effect of between-subject differences in positive symptoms. Thus, higher scores in positive symptoms across assessment times were significantly associated with higher OCS severity across assessments. Furthermore, within-subject variability in all three CAPE variables remained significantly associated with OCS severity. Random effects still showed significant heterogeneity in intercept and within-subject variation (Table 2).

**Table 2:** CAPE symptom clusters as predictors of OCS in patients

Fixed effects	Estimate (SE)	*t*	*p*
Intercept	2.50 (1.46)	1.71	.094
Between-subject			
CAPE_positive	0.79 (0.13)	6.09	< .001
CAPE_negative	0.20 (0.22)	0.93	.358
CAPE_depressive	0.25 (0.41)	0.61	.543
Within-subject			
CAPE_positive	0.24 (0.11)	2.27	.024
CAPE_negative	0.27 (0.08)	3.27	.001
CAPE_depressive	0.36 (0.14)	2.67	.008
Time	− 0.91 (0.64)	− 1.42	.161

Random effects	Estimate (SE)	*z*	*p*
Intercept	32.13 (7.44)	9.99	< .001
Time (slope)	5.12 (4.22)	1.21	.225
Covariance (Intercept*slope)	0.40 (4.22)	0.09	.925
Level 1 (within-person)			
Residual	9.99 (1.00)	9.99	< .001
− 2LL	1769.64		

*SE* standard error, *− 2LL* − 2 Log Likelihood

To account for possible confounding effects, we added gender, illness duration, IQ estimate, and type of antipsychotic medication as covariates to the model. None of these variables significantly contributed to the analyses and did not change the results.

To further investigate the quantitative effect of CAPE contribution to the variability in OCS, effect sizes were calculated based on *R*^2^. Therefore, the residual variance of the model with no predictors was compared to the residual variance of the full model including all predictors [28]. Compared with a baseline model, residual variance was reduced by 18%, whereas random intercept variance was reduced by 72% when within-subject and between-subject scores were added in a model, where CAPE predicted OCS.

Within siblings, mixed-model analyses revealed significant associations between OCS and between-subject differences in CAPE positive (*T* = 3.68, *p* = .001), negative (*t* = 2.63, *p* = .011), and depressive symptoms (*t* = 3.60, *p* = .001) and within-subject variability in these three domains (positive: *t* = 3.20, *p* = .002, negative: *t* = 2.28, *p* = .024, and depressive symptoms: *t* = 3.19, *p* = .002).

Analyses of a full model including all predictors resulted in a significant effect of between-subject differences and within-subject variability in positive symptoms. Within-subject variability in depressive symptoms also reached significance. Again, random effects still showed significant heterogeneity in intercept and within-subject variability (Table 3). Adding gender and IQ estimate to the analyses as potential confounders did not change significant associations with positive symptoms. Associations with within-subject variability in depressive symptoms were reduced to a trend (*T* = 1.82, *p* = .071).

**Table 3:** CAPE symptom clusters as predictors of OCS in siblings

Fixed effects	Estimate (SE)	*t*	*p*
Intercept	1.94 (0.65)	2.99	.004
Between-subject			
CAPE_positive	1.25 (0.47)	2.67	.011
CAPE_negative	− 0.02 (0.19)	− 0.12	.905
CAPE_depressive	0.46(0.27)	1.69	.098
Within-subject			
CAPE_positive	0.39 (0.17)	2.32	.021
CAPE_negative	0.02 (0.07)	0.31	.757
CAPE_depressive	0.20 (0.10)	1.95	.052
Time	− 1.20 (0.43)	− 2.77	.008

Random effects	Estimate (SE)	*z*	*p*
Intercept	7.63 (1.95)	3.92	< .001
Time (slope)	4.38 (184)	2.37	.018
Covariance (Intercept*slope)	− 2.84 (1.54)	− 1.84	.066
Level 1 (within-person)			
Residual	2.78 (0.29)	9.47	< .001
− 2LL	1252.59		

*SE* standard error, *− 2LL* − 2 Log Likelihood

Compared with a baseline model including only the random effects (intercept, time), residual variance was reduced by 8%, whereas random intercept variance was reduced by 21% when within- and between-subject scores were added in a model, where CAPE predicted OCS.

### Reciprocal associations in the prediction of symptom severity 4 weeks later

We first investigated these associations in patients. With OCS 4 weeks later as the dependent variable, between-subject differences in positive (*t* = 8.69, *p* < .001), negative (*t* = 3.36, *p* = .001), and depressive symptoms (*t* = 4.64, *p* < .001) were identified as significant predictors, whereas only within-subject variability in depressive symptoms reached significance (*t* = − 2.08, *p* = .039). When including significant predictors in one model, only between-differences in positive symptoms (*t* = 6.26, *p* < .001) and within-subject variability in depressive symptoms (*t* = − 2.08, *p* = .039) remained significant. Including confounders in the analysis did not significantly change the results.

To investigate possible reciprocal relationships, we included between-subject and within-subject variability in OCS as the predictor of CAPE symptom domains. Between-subject differences in OCS significantly predicted positive symptoms (*t* = 8.45, *p* < .001), negative symptoms (*t* = 3.42, *p* = .001), and depressive symptoms (*t* = 4.59, *p* < .001) 4 weeks later. Within-subject variability in OCS did not significantly predict positive, negative, or depressive symptoms.

In siblings between-subject differences in positive (*t* = 3.46, *p* = .001), negative (*t* = 3.11, *p* = .003) and depressive symptoms (*t* = 3.62, *p* = .001) were identified as significant predictors, with OCS 4 weeks later as the dependent variable. No within-subject variability significantly predicted subsequent OCS severity in siblings. When including significant predictors in one model, only between-differences in positive symptoms (*t* = 2.56, *p* = .014) remained significant, even after including IQ and gender as potential confounders.

Regarding reciprocal relationships, between-subject differences in OCS significantly predicted positive symptoms (*t* = 2.56, *p* = .014), negative symptoms (*t* = 6.40, *p* = .015), and depressive symptoms (*t* = 2.97, *p* = .005) 4 weeks later. Again, within-subject variability in OCS did not significantly predict positive, negative or depressive symptoms.

## Discussion

The aim of the current study was to investigate the course of obsessive–compulsive, positive, negative, and depressive symptoms over a 6 month period and specifically focus on within-subject changes and possible co-variation of the different symptom domains.

High variability between-subjects in the initial severity of symptoms was found for all domains in patients and on a subclinical level in siblings. Psychotic positive and depressive symptoms varied markedly over time across individuals in patients and siblings. Siblings also showed significant variance in rate of change in subclinical OCS and negative symptoms. Apart from these expected between-subject differences, analyses further revealed substantial within-subject variability in all symptom domains in patients (Fig. 1) and on a lower level in siblings (supplement Fig. 1).

Mixed-model analyses investigating the disaggregated effects of between-subject differences and within-subject changes in severity of positive, negative, and depressive symptoms on OCS severity showed overall association between severity of symptom clusters across patients and siblings. Hence, higher severity of positive, negative, and depressive symptoms was closely associated with higher concurrent OCS severity across subject and assessment time and significantly predicted OCS severity 4 weeks later. Vice versa between-subject differences in OCS predicted subsequent symptoms of psychosis. When including all predictors in one model, only between-subject differences in the severity of positive symptoms remained significant in the prediction of concurrent and subsequent OCS. Regarding the main focus of interest, changes within individuals showed concurrent co-variation of OCS severity and positive, negative, and depressive symptoms in patients and siblings. In patients, within-subject variation in all three symptoms domains added to the full model, demonstrating specific contribution to explained variance in OCS severity. Regarding the prediction of OCS 4 weeks later, only within-subject variation in depressive symptoms in patients showed a significant effect, which independently contributed to a full model. Hence, an increase/decrease of a patient’s depressive mood predicted subsequent reduction/increase in OCS severity. This association was not found the other way around.

Results stand in line with longitudinal studies reporting significant fluctuation in co-occurring symptoms over time [8, 15]. Our data extends the literature by frequent self-rated assessments, which show that variability in symptoms can be observed within several months and occur across and within individuals in patients and on a subclinical level in siblings. As described by several studies, our findings replicate associations between symptom dimension of psychosis and OCS [7, 10, 15]. Results further show the importance to disaggregate between-subject and within-subject effects, because the relationship between symptom clusters seems to differ at these two levels. Whereas findings highlight the strong association between positive symptoms and OCS across subjects and timepoints, self-rated depressive symptoms seem to be more closely related to OCS within patients and on a subclinical level within siblings. This also holds true for negative symptoms within patients.

Associations on the between- and within-subject level may be due to different factors, which affect the co-occurrence. Regarding the associations of OCS and positive symptoms on the between-subject level, common pathogenic mechanisms have been proposed. Familial aggregation of schizophrenia–spectrum disorders and OCD and cross-sib, cross-trait associations support a shared genetic vulnerability [14, 29, 30]. Common neurobiological mechanisms and overlapping neural network representations have further been described for both conditions [31, 32]. In addition, personality traits, specifically high neuroticism, have been linked to psychotic symptoms, obsessive–compulsive symptoms, and their co-occurrence in patients and siblings [33–36].

Concurrent co-variation within individuals on the other hand could depend on overlapping time-varying factors, for example the experience of daily hassles or interpersonal conflict, which cause simultaneous increase or decrease of symptom severity. However, to the best of our knowledge, the influence of these environmental factors on co-variation of OCS and psychosis has not yet been investigated. Within-subject associations might further be due to symptom interaction, where increase in one type of symptom causes the other [37, 38]. This proposed mechanism might specifically apply to the observed unidirectional time-lagged association between increase/decrease in depressive symptoms and subsequent increase/decrease in OCS 4 weeks later. Comparable associations have been reported before. On a broader level, we recently found higher pre-existing emotional distress in a group, which developed OCS 3 years later [15]. Within primary OCD, Rickelt et al. reported that depressive symptoms at baseline predicted OCS severity 1 year later but not vice versa [39]. A proposed underlying mechanism suggests that depressive affect frequently goes along with rumination, worries, and doubt, which are strongly related to obsessive thoughts and might be associated with a subsequent attempt to reduce resulting anxiety through compulsive behaviour. McNally and colleagues examined the potential causal relationships among symptoms of OCD and depression in a recent network analysis and found sadness and anhedonia to be the central nodes in linking obsessive–compulsive and depressive symptoms [40]. The observation that these mechanisms might be specifically relevant within-individuals has important treatment implications. Assessing and addressing negative affect and depressed mood in our patient sample might lead to subsequent reduction or prevention of co-occurring OCS.

Noteworthy, large heterogeneity in between- and especially within-subject variability of OCS remained unexplained, suggesting that other variables cause significant symptom fluctuation and possible co-variation. As hypothesized, these might include environmental (e.g., daily life events, pharmacotherapy) or individual characteristics (e.g., coping skills, fatigue) as well as interactions between them. Furthermore, assessment intervals of 4 weeks might still be too long to detect within-subject causal interrelations. Future studies should involve Experience Sampling Methods (ESM), daily investigation in real life situations, which allow detecting time sequences and predictors of changes in symptom severity on a real-time level [41]. New developments in network analyses incorporate a temporal dimension might be another interesting approach to understand how symptom interaction unfolds over time [42].

Specific limitations should be acknowledged. Due to the observational nature of our study, the discussion of possible mechanisms and causal interactions remains speculative. Furthermore, participants of the GROUP study represent a relatively high functioning group with mild to moderate symptom severity, which limits the generalizability of our result. Between-subject and within-subject associations in patients with more severe co-occurring OCS and psychosis might differ and should be investigated in future studies. Finally, severity of symptoms was assessed with self-report instruments. Although these have been found valid in assessing psychotic and obsessive–compulsive symptoms, findings should be replicated with interview-based outcome measures.

In conclusion, this is the first prospective study investigating between-subject and within-subject associations of OCS and symptom clusters of psychosis in patients with psychotic disorders and their un-affected siblings. Findings indicate that especially severity of positive symptoms is strongly related across individuals and time to OCS in patients and siblings, suggesting shared underlying vulnerability. Furthermore, a specific focus on within-subject variability is necessary to understand interrelations of symptom dimensions. Especially, the associations between depressive symptoms and subsequent change in OCS severity 4 weeks later may have important treatment implications. Further studies are needed to replicate found associations and to investigate proposed overlapping time-varying factors, which may cause concurrent symptoms co-variation within-individuals.

## Electronic supplementary material

Below is the link to the electronic supplementary material.

Supplementary material 1 (PDF 382 KB)
