# Supplementary material for: Obsessive–compulsive symptoms in psychotic disorders: longitudinal associations of symptom clusters on between- and within-subject levels
Source: Eur Arch Psychiatry Clin Neurosci. 2018 Mar 8;269(2):245–55. doi: 10.1007/s00406-018-0884-4 (PMC6726663; doi:10.1007/s00406-018-0884-4)
Supplement: Supplementary file 1 — Supplementary material 1 (PDF 382 KB) [file 406_2018_884_MOESM1_ESM.pdf]

Figure 1 Supplement

Within-subject change in obsessive-compulsive, positive, negative and depressive symptoms over 6 months in siblings.

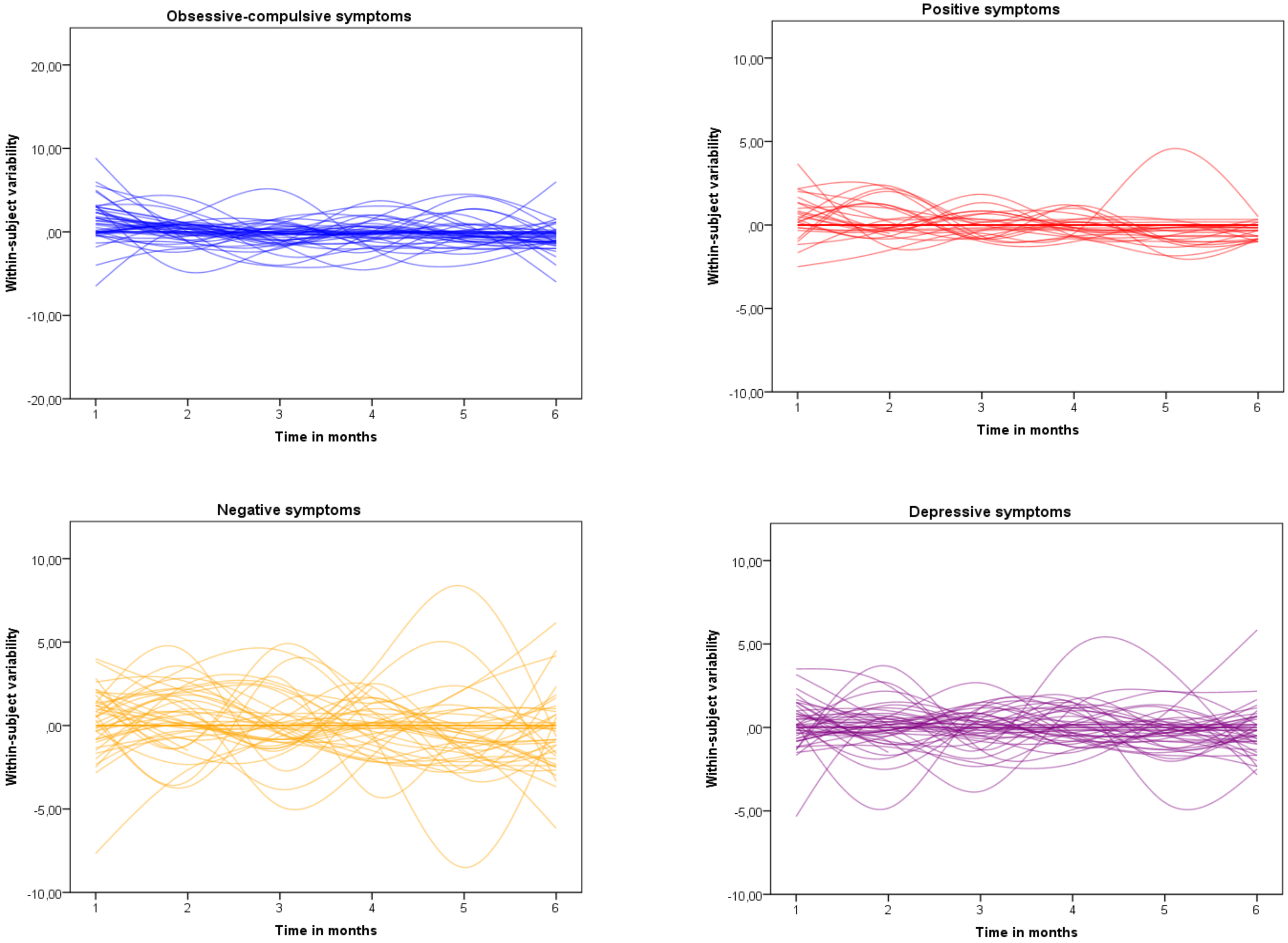

Legend: ----- obsessive-compulsive (in blue), -----positive (in red), -----negative (in orange), -----depressive (in purple)

Figure 2 Supplement
Examples of 8 individual panel plots of within-subject (co-)variation in symptom severity in siblings.

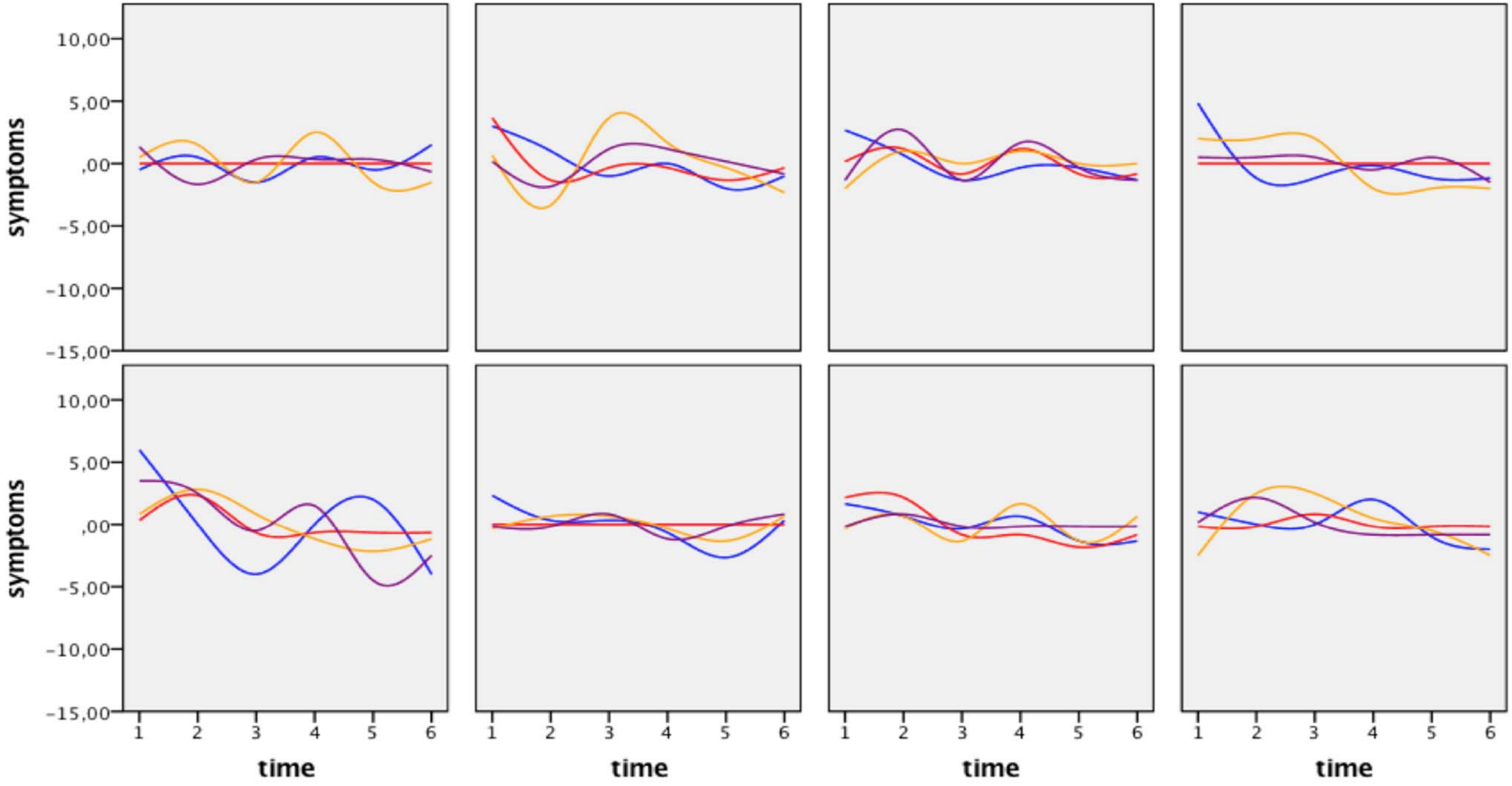

Legend: ----- obsessive-compulsive (in blue), -----positive (in red), -----negative (in orange), -----depressive (in purple)
